# Extreme vulnerability of smallholder farmers to agricultural risks and climate change in Madagascar

**DOI:** 10.1098/rstb.2013.0089

**Published:** 2014-04-05

**Authors:** Celia A. Harvey, Zo Lalaina Rakotobe, Nalini S. Rao, Radhika Dave, Hery Razafimahatratra, Rivo Hasinandrianina Rabarijohn, Haingo Rajaofara, James L. MacKinnon

**Affiliations:** 1 Conservation International, 2011 Crystal Drive Suite 500, Arlington, VA 22202, USA; 2 Conservation International Madagascar, Villa Hajanirina, lot II W 27D, Rue Vittori Francois, Ankorahotra, 101 Antananarivo, Madagascar; 3 High School of Agricultural Sciences, University of Antananarivo, PO Box 175, Antananarivo, Madagascar; 4 IDACC Association, Lot VS 52D, Bis Ankatso Nord Antananarivo 101, Madagascar

**Keywords:** adaptation, agriculture, climate change, food security, livelihoods, Madagascar

## Abstract

Across the tropics, smallholder farmers already face numerous risks to agricultural production. Climate change is expected to disproportionately affect smallholder farmers and make their livelihoods even more precarious; however, there is limited information on their overall vulnerability and adaptation needs. We conducted surveys of 600 households in Madagascar to characterize the vulnerability of smallholder farmers, identify how farmers cope with risks and explore what strategies are needed to help them adapt to climate change. Malagasy farmers are particularly vulnerable to any shocks to their agricultural system owing to their high dependence on agriculture for their livelihoods, chronic food insecurity, physical isolation and lack of access to formal safety nets. Farmers are frequently exposed to pest and disease outbreaks and extreme weather events (particularly cyclones), which cause significant crop and income losses and exacerbate food insecurity. Although farmers use a variety of risk-coping strategies, these are insufficient to prevent them from remaining food insecure. Few farmers have adjusted their farming strategies in response to climate change, owing to limited resources and capacity. Urgent technical, financial and institutional support is needed to improve the agricultural production and food security of Malagasy farmers and make their livelihoods resilient to climate change.

## 1. Introduction

Smallholder farmers constitute a significant portion of the world's population, with an estimated 450–500 million smallholder farmers worldwide, representing 85% of the world's farms [1]. Smallholder farmers are also estimated to represent half of the hungry worldwide and probably three-quarters of the hungry in Africa [2]. Consequently, the fate of smallholder farmers will largely determine whether or not the world succeeds in reducing poverty and hunger worldwide and meeting the Millennium Development Goals.

Across the tropics, smallholder farmers already face numerous risks to their agricultural production, including pest and disease outbreaks, extreme weather events and market shocks, among others, which often undermine their household food and income security [3,4]. Because smallholder farmers typically depend directly on agriculture for their livelihoods and have limited resources and capacity to cope with shocks, any reductions to agricultural productivity can have significant impacts on their food security, nutrition, income and well-being [5,6].

Climate change is expected to disproportionately affect smallholder farmers by further exacerbating the risks that farmers face. Recent studies using regional and global simulation models, for example, indicate that even moderate increases in temperatures will have negative impacts on rice, maize and wheat, which are the main cereal crops of smallholder farmers [4]. Climate change is also expected to alter pest and disease outbreaks, increase the frequency and severity of droughts and floods, and increase the likelihood of poor yields, crop failure and livestock mortality [4,7]. As many of the countries that will be the hardest hit by climate change are tropical countries with large populations of poor, smallholder farmers [5], there is an urgent need for the global community to focus its attention on identifying adaptation measures that can help these farmers reduce their vulnerability to climate change and cope with adverse consequences.

Madagascar is a country in which understanding the vulnerability of farmers to agricultural risks and climate change is particularly important, as farmers comprise approximately 70% of the population [8] and climate change impacts are expected to be significant [9]. Madagascar has one of the highest poverty rates in Africa, with 81% of the island's inhabitants living on less than the international poverty threshold of $1.25 per day (PPP) and *per capita* gross national income (GNI) being just $430 [10]. In 2011, Madagascar was ranked 151 out of 187 countries assessed for the Human Development Index [11]. An estimated two-thirds of the Malagasy population is considered undernourished [12] and 82% of the rural population falls below the national poverty line [13]. Most farmers are smallholders (with a national average upland rice area per farmer of 1.28 ha, [14]), cultivate primarily for subsistence, are chronically food insecure, and generally lack basic services, such as improved water sources and electricity [15]. Madagascar has suffered significant deforestation and forest fragmentation over the last 50 years (in large part owing to agriculture), with the forest cover decreasing almost 40% from the 1950s to 2000 and much of the remaining forest land being highly degraded [16]. In addition, much of the agricultural land is severely eroded owing to unsustainable land-use practices [17].

While several studies have characterized the livelihoods of Malagasy farmers and explored factors influencing poverty and food insecurity [8,12,14,18,19], there is limited information on the overall vulnerability of farmers to different agricultural risks (both climate and non-climate related) and the strategies that farmers use to cope with these risks. In addition, there is little information on what adaptation measures are needed to reduce farmer vulnerability in the context of climate change. Madagascar is already subjected to periodic extreme weather events, including cyclones, flooding and droughts, and it is expected that these events will intensify under climate change [9].

In this study, we explore the vulnerability of smallholder farmers to agricultural risks in Madagascar and provide recommendations on which risk management and adaptation strategies hold the greatest potential for reducing farmer vulnerability. Specifically, we characterize the vulnerability of smallholder farmers to different risks (both climate and non-climate related), identify the risk coping and adaptation strategies used by farmers, and highlight key adaptation needs. By increasing knowledge of the impacts of risks to agriculture and the existing coping strategies that farmers use, our study provides critical information for development organizations and donors focused on food security and poverty alleviation in rural areas of Madagascar, as well as for policymakers working on the design of both national and international strategies for climate change adaptation, agricultural productivity, and hunger and poverty alleviation.

## 2. Material and methods

**Figure 1.**
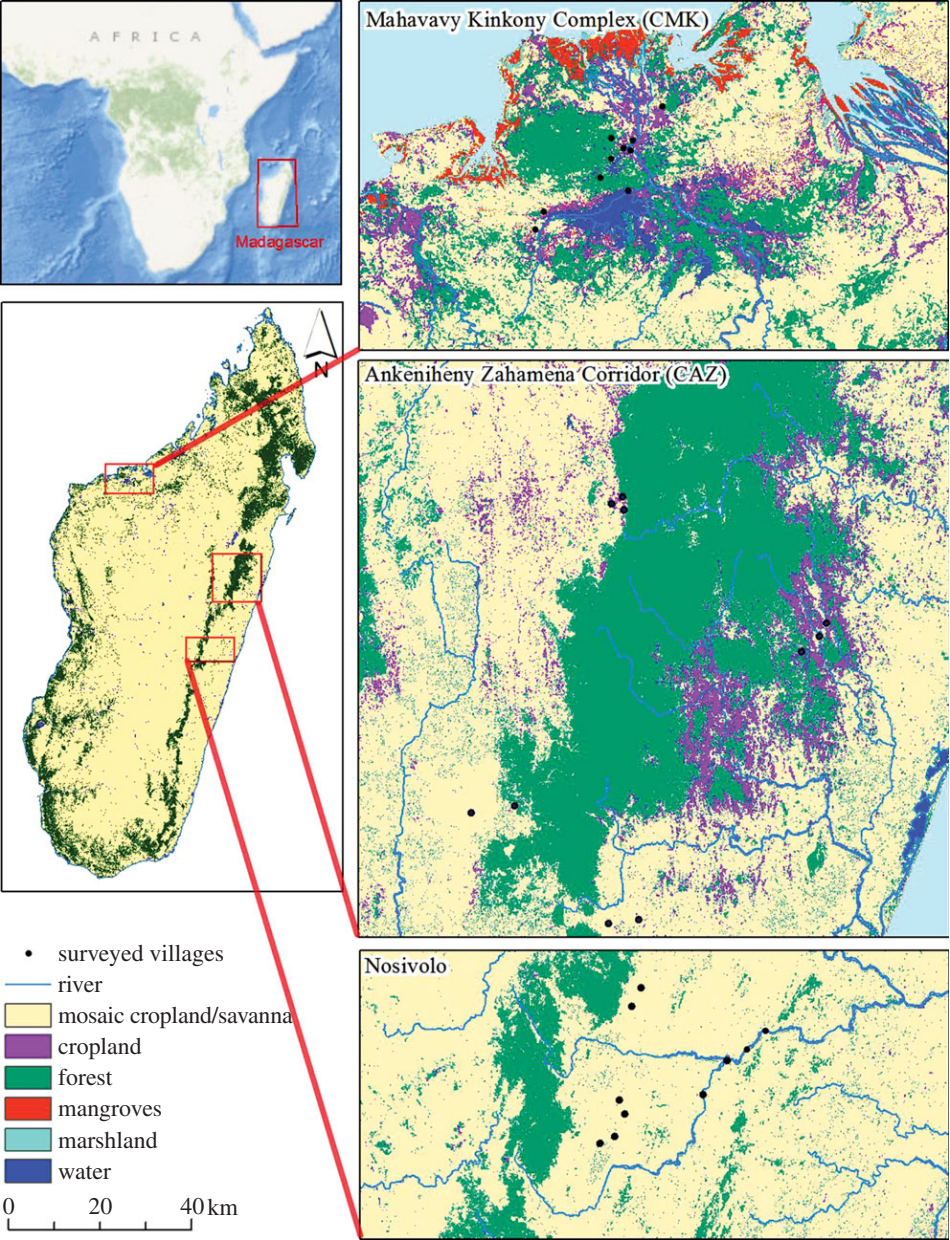
Map showing the location of the three study landscapes, the key land uses in each landscape and the location of the 10 villages per landscape (30 total), where household surveys were conducted. (Online version in colour.)

We assessed farmer vulnerability to agricultural risks in three different landscapes of Madagascar: Ankeniheny-Zahemena Corridor (French acronym: CAZ), Nosivolo (NSV) and Mahavavy Kinkony Complex (French acronym: CMK; figure 1). Both CAZ and NSV are located around Madagascar's eastern escarpment and are characterized by a moist, subtropical climate. The Ankeniheny-Zahamena Corridor has one of the largest remnants of tropical rainforest in eastern Madagascar, surrounded by agricultural land. The forest is in the process of being formally gazetted as a protected area [20–22]. The Nosivolo landscape is a diverse mosaic of agricultural land and patches of tropical rainforest in the watershed of the Nosivolo River [23]. CMK is a complex of lakes, wetlands and agricultural land, with a small area of remaining tropical dry forest in northwestern Madagascar, which experiences a dry and warm climate, with two distinct seasons [24]. In all three sites, farming is centred on rice, cassava and maize production. In CAZ and NSV, rice production on the hillsides is mainly rain fed and done using slash and burn (‘tavy’), while rice in the lowland, flatter areas of these landscapes and in most of CMK is produced in paddy fields. Our study landscapes are representative of three of the four major agroecological regions (the western region, highlands and the eastern region) present in the country, but do not cover the drier southern ecosystem.

We characterized farmer vulnerability to agricultural risks using detailed household surveys and focal group discussions. In each landscape, we first met with key informants (e.g. local non-governmental organizations, mayors, chiefs of fokontany—the smallest administrative unit in Madagascar comprising one or a couple of villages—and village chiefs) to describe the project, obtain input into the proposed research, identify the main farming systems present and begin discussion of the risks affecting agricultural production. Using this information, we selected 10 villages per landscape that were considered representative of the main farming systems present, and then randomly selected 20 households per village for interviews (i.e. 200 households per landscape and 600 households in total).

The household survey was designed to characterize farmer livelihood strategies, agricultural risks, risk coping mechanisms, perceptions of climate change and adaptation needs. Prior to applying the surveys, we reviewed the survey questions, content and terms with focal groups, and tested the survey with 20 randomly selected farmers. The final survey consisted of 197 questions, most of which were multiple choice or close-ended questions. We conducted the household surveys during a six-week period from November to December 2011. In each region, the survey team consisted of a lead, together with 10 locally recruited interviewers (who were native speakers of Malagasy and trained on sample design and survey techniques). The interviews were conducted in Malagasy at the participant's home or farmland, and took approximately 1 h and 45 min to complete. In each household, we conducted interviews with the self-identified head of the household (usually a man). All information from the interviews was recorded manually on data sheets by the interviewers and checked for accuracy by lead field research staff. In addition, survey results were discussed with focal groups in each landscape (consisting of 10 men and 10 women) to ensure accurate interpretation of results. In the household survey, response rates of participants were high (usually more than 98%), but because the structure of the survey allowed household members to skip certain sections of the survey if these sections did not apply to the household or if they had answered in a certain way to a previous question, the sample size per answer varied and is therefore noted in the tables.

All data were analysed using either Infostat v. 2012 [25] or Stata [26]. All data analyses were conducted on the combined data from the three landscapes. To explore which factors influenced household risks relevant to food security, we developed indices of key variables of household and farm characteristics (scaled from zero to one) and then ran Spearman correlations of each index against our variable of household food insecurity (= the number of months in which farmers reported not having enough rice from their own production to feed their household in a typical year). We similarly used Spearman correlations to explore relationships between household and farm characteristics versus the number of adaptation measures each farmer had adopted.

## 3. Results

### (a) Household characteristics

Across the three regions, the living conditions of smallholder farmers were poor, with farmers living in small, basic houses made of local materials (*Raphia ruffia*, bamboo*, Ravenala madagascariensis* and/or mud) and most households (98%) depending on firewood for cooking and oil for light. Nearly half of the farmers obtained their water directly from rivers and an additional 17% from lakes or ponds; only 13% had access to public taps. Smallholder farmer education levels were low, with 27% of the farmers lacking any formal education and an additional 48% having only completed primary school. Seventy-one per cent of the smallholder farmers were born in the villages where they currently live; the remainder were migrants who had moved to the villages either to improve their standard of living or because of marriage. Most smallholder farmers lacked a means of transportation (only 15% owned bikes and 13% owned oxcarts), so have to walk, often several hours, to get their products to market. Just under a fifth of all households had access to mobile phones. Less than 2% of farmers had personal saving accounts or were members of a village savings account. Mean household size was 7.5 (±0.1) members.

### (b) Farming systems

Farmers typically had several plots of land, with some under tavy (slash and burn) for rice production, some plots in lowlands or wet areas for rice production, and others dedicated to other agricultural crops, such as cassava, maize, vegetables or fruits (table 1). In most cases, the plots of land were small, with 68% of farmers having less than 1 ha under tavy for rice production and 32% having less than 200 m^2^ under tavy. Rice, cassava and maize were the most common crops, cultivated by 89%, 91% and 72% of all farmers, respectively, but a subset of farmers (particularly in CAZ and NSV) also cultivated additional crops, such as bananas, beans, sweet potatoes and others. In addition, many farmers had small numbers of livestock, particularly chickens, and also cattle, pigs and ducks. Farmers used low technology management approaches, such as intercropping (e.g. maize/beans and rice/maize), and to a lesser degree agroforestry systems and fire (particularly in tavy systems). Few farmers practised soil conservation techniques, despite the steep slopes present. The use of agricultural inputs, chemical fertilizers, and improved seed varieties was very low, and only 7% of farmers reported receiving any technical assistance on crop or livestock production.

**Table 1.** Characteristics of smallholder farming systems in three regions of Madagascar based on household surveys. Data represent the per cent of households or the means and standard errors across households.

category	*N*	variable	% of households
total area under tavy for rice production	505	<200 m^2^	32
		200–500 m^2^	14
		500 m^2^–1 ha	22
		1–2 ha	17
		>2 ha	15
total area under non-tavy rice production	565	<200 m^2^	28
		200–500 m^2^	16
		500 m^2^–1 ha	27
		1–2 ha	18
		>2 ha	11
total area under other agricultural systems	527	<200 m^2^	41
		200–500 m^2^	20
		500 m^2^–1 ha	23
		1–2 ha	11
		>2 ha	5
crops grown (ordered from the most common overall to the least common)	600	cassava	91
		rice	89
		maize	72
		bananas	53
		beans	49
		sugarcane	48
		sweet potatoes	36
		peanuts	25
		taro	25
		coffee	24
		litchi	24
		oranges	22
		ginger	14
		mangos	14
		potatoes	10
household crop diversification	600	mean number of crops per household	6.0 (+0.14)
use of specific agricultural practices (in decreasing order of importance)	597	intercropping	43
		fire	38
		multiple cropping	37
		irrigation	25
		biological control	23
		manure fertilizer	22
		agroforestry	14
		improved seed varieties	12
		chemical fertilizer	9
		soil conservation practices	6
livestock ownership	600	chickens	71
		cattle	38
		pigs	21
		ducks	11
		goats	1
technical assistance for crop or livestock production	598		7

### (c) Livelihood strategies and food security

Agriculture was the primary livelihood activity for households in all three regions in both seasons (table 2), but particularly in the dry season when it provides more than 50% of all of their income. Other common sources of income included livestock production (79% of farmers) and occasional outside work (43%), primarily as agricultural labourers on other farms. In all three regions, rice and cassava (and maize in CMK) were the most important crops both for home consumption and income generation. For example, more than 85% of all households reported consuming half or more of their rice harvests, and of these 45% consume more than three-quarters of their rice harvests.

**Table 2.** Household livelihood strategies and food security of smallholder farmers in Madagascar based on 600 household surveys. Number represents the per cent of households or the mean number per household.

	variable	*N*	variable	total
livelihood strategies	sources of household income (ordered from the most common overall to the least common)	600	agriculture	99
			livestock	79
			occasional work off-farm	43
			handicrafts	19
			fishing	14
			commerce (small scale)	10
			mining	8
			salaried permanent work	5
			charcoal production	3
			logging	2
	income diversification	600	no. of sources of income per household	2.8 ± 0.04
	households selling staple crops	533	rice	84
		473	cassava	87
		432	maize	61
	per cent of household income derived from agriculture during the wet season	589	<25%	27
			25–50%	41
			50–75%	22
			>75%	10
	per cent of household income derived from agriculture in dry season	588	<25%	17
			25–50%	22
			50–75%	40
			>75%	21
food security	per cent of rice production used for home consumption	529	<25%	3
			25–50%	12
			50–75%	40
			75%	45
	per cent of cassava production used for home consumption	508	<25%	7
			25–50%	37
			50–75%	31
			>75%	25
	per cent of maize production used for home consumption	362	<25%	6
			25–50%	20
			50–75%	29
			>75%	45
	household food insecurity	600	% of households who do not produce sufficient food to feed their households year-round (in a typical year)	75
		600	mean number of months that households lack sufficient food in a typical year	3.8 ± 0.1

**Figure 2.**
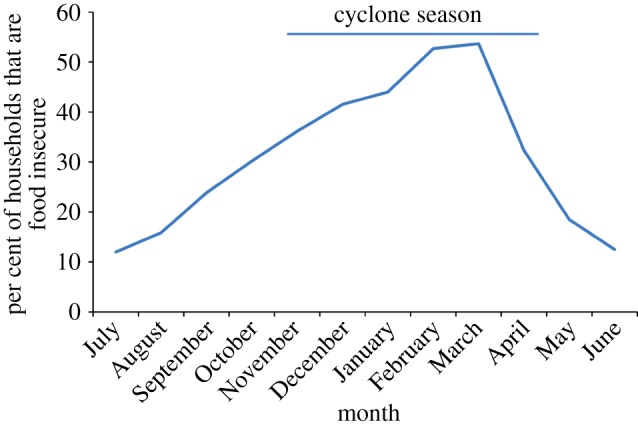
Seasonal pattern of food insecurity among smallholder farmers in Madagascar in a regular year. Data show the per cent of farmers (*n* = 600) who reported having insufficient food at different times of the year. The line above the graph represents the typical cyclone season in Madagascar and indicates the overlap between periods of food shortages and the occurrence of cyclones.

Food security was a significant problem for farmers, with 75% of the households reporting that they did not produce sufficient rice to feed their households year-round. In each of the regions, farmers reported a distinct ‘lean’ season (from December to March) during which more than 40% of the households lacked sufficient food (figure 2). This lean season occurs at the beginning of the rainy season before the rice harvest and extends for an average of 3.8 months. There was great variation across households in the level of food insecurity, however, with 27.3% lacking sufficient food for six months or more each year and 5.5% of the population suffering food insecurity year-round. Factors that were positively related to household food security included livestock ownership (*r* = 0.10, *p* = 0.01), ownership of means of transportation (e.g. either an oxcart or a bicycle; *r* = 0.15, *p* < 0.001), household head being born in the village (*r* = 0.46, *p* < 0.01) and higher education level of the household head (*r* = 0.64, *p* < 0.0001).

### (d) Risks to agriculture and farmer livelihoods

Smallholder farmers faced frequent risks to their agriculture, including disease outbreaks, pest damage, crop loss during storage and occurrence of extreme weather events (table 3). The amount of crop lost to pests, diseases, storage problems or extreme weather events—and the accompanying income loss—was highly variable across households, with impacts ranging from mild to severe. For example, during the last cyclone 29% of farmers lost less than a quarter of their crops to the cyclone, while 10% lost more than 75% of their crops. The prevalence of extreme events was particularly notable: in the last 5 years, cyclones have affected 51% of all farmers surveyed, while severe drought and flooding have affected 68% and 44%, respectively. Extreme weather events were reported to have significant impacts on farmer food security, increasing the number of months in which they lack sufficient food. On average, households experienced 3.8 (±0.1) months of food insecurity following cyclones, 3.4 (±0.1) months following floods and 3.2 (±0.1) months following droughts. Following the last cyclone, 89% of farmers had to rebuild the roofs or walls of their houses.

**Table 3.** Summary of the risks to rice production experienced by smallholder farmers and the impacts of these risks on rice yields and household income (as reported by farmers). Numbers represent the per cent of farmers experiencing this problem or the means (+s.e.).

agricultural risk	*n*	% of farmers affected	frequency of risks (mean number of occurrences in last 5 years)	% of crop yields lost due to risks				% reduction in household income due to risk			
				<25%	25–50%	50–75%	>75%	<25%	25–50%	50–75%	>75%
significant disease outbreak	539	47	1.6 (± 0.08)	56^a^	29^a^	15^a^	—	10^a^	32^a^	41^a^	15^a^
severe pest damage	539	81	3.1 (± 0.09)	—	—	—	—	—	—	—	—
loss of crops during storage	539	36	1.3 (± 0.09)	88	10	2^b^	—	—	—	—	—
cyclones	524	51	1.2 (± 0.1)	30	29	30	26	39	30	21	10
severe flooding	524	44	1.2 (± 0.1)	40	35	20	5	40	34	17	8
severe drought	524	68	1.8 (± 0.1)	23	42	27	9	35	35	22	8

^a^Impacts of pests and diseases on crop yields and income levels were assessed jointly, owing to difficulties of attributing impacts to one or the other.

^b^These numbers (for crop storage) refer to losses of more than 50%.

Farmers also faced risks to their livestock, including disease (affecting 14% of households), theft (7%) and drought (2%), though the per cent of households affected by these risks was much smaller than that of households experiencing risks to agriculture. Other risks to farmer livelihoods were related to market issues, such as high volatility in market prices for agricultural products (reported by 90% of farmers), large increases in the prices of agricultural inputs (60%), low prices for their products (58%) and problems getting products to markets owing to impassable roads (37%).

### (e) How farmers cope with food insecurity and income loss

Farmers reported using a variety of coping strategies to deal with food insecurity. When farmers experience food shortages, they respond by eating smaller meals, eating fewer times a day, changing their diet (principally from rice to cassava or maize; table 4) or supplementing their food supplies by harvesting wild yams (*Dioscorea* species) and other tubers in nearby forests. Farmers also supplement their food supplies by purchasing rice from market and routinely sell household assets (particularly chickens) or send household members to get outside employment (as an agricultural labourer on another farm) to obtain income to buy food. Social networks were also critical, with 20% of households indicating that they borrowed money from friends, neighbours or local organizations to buy food, and 10% of families receiving food from neighbours or relatives. Interestingly, only 1% of the farmers reported receiving food aid from local institutions. Farmers also reported helping each other to collect raw materials (palms, bamboo and other plants for thatch and timber) and rebuild damaged houses following cyclones.

**Table 4.** Per cent of households using different coping strategies to deal with reduced agricultural production, food insecurity and income loss in three regions of Madagascar (ordered from most to least common). Sample size ranged from 596 to 600 households per question.

coping strategies	total
ate less food	81
reduced number of meals/day	60
purchased food	67
changed diet	51
sold assets to buy food	42
borrowed money	20
received food from relatives	16
increased consumption of wild plants and animals	14
sent older children away to work	9
received food from neighbours/community	8
took boys out of school	7
took girls out of school	6
made children work more on the farm	6
sent an adult household member to get an outside job	6
leased their land to other farmers	1
received food aid from organization	1

### (f) Adaptation strategies for extreme weather events and climate change

There was a general perception among the smallholder farmers that climatic conditions have changed over the last 10 years. Commonly observed climatic trends included higher temperatures (reported by 95% of farmers), lower rainfall (94%), more variable rainfall (95%), greater variability in seasons (89%) and stronger cyclones (58%). Only a subset of farmers reported having made changes in their farming practices to either reduce their future vulnerability to droughts and floods or to accommodate long-term shifts in climatic conditions (table 5). The most common adaptation strategies ranged from planting new crops or new varieties, to better water management, to the implementation of practices (e.g. soil conservation practices) to improve agricultural sustainability, to measures for managing water resources. However, the perceived effectiveness of these strategies was very low, with roughly 50% of all farmers indicating that their adaptation strategies for drought and flooding were not effective. The number of adaptation measures adopted per household was positively correlated with greater sources of income (*r* = 0.25, *p* < 0.001), farmer education levels (*r* = 0.19, *p* < 0.001), being born in the village (*r* = 0.16, *p* < 0.001), the diversity of crops (*r* = 0.4, *p* < 0.001) and management techniques used on farm (*r* = 0.46, *p* < 0.001) and livestock production (*r* = 0.16, *p* < 0.01), and negatively correlated with household food insecurity (*r* = 0.09, *p* = 0.03).

**Table 5.** Management practices that smallholder farmers have put in place to decrease their vulnerability to drought, flooding and changing climatic conditions. Percentages refer to the per cent of those farmers who made this change in response to a given risk.

agricultural risk	*n*	types of changes made by farmers in response to different risks	% of farmers
drought	432	changed timing of crop planting	28.2
		changed crops grown	16.0
		changed crop varieties	9.3
		changed location of crop fields	7.2
		built a water-harvesting system for crops	3.7
		installed an irrigation system	2.1
flooding	297	replanted crops after flooding subsided	22.2
		built diversion ditches to remove water from fields	16.8
		changed timing of crop planting	11.1
		changed crop varieties	10.1
		stopped farming the land that was flooded	9.4
		changed crop type	8.4
climate change (generally)	543	increased use of intercropping	22.5
		built a communal granary or food storage system to store crops	18.8
		changed the location of fields	15.1
		diversified production system by incorporating trees	13.1
		implemented soil and water conservation practices	11.2
		changed crop varieties	11.0
		changed type of crop	9.6
changes in water availability owing to climate change	544	built ditches to direct water or floods away from certain areas	18.2
		developed irrigation system for crops	11.6
		built a water-harvesting scheme for crops	8.2
		built a water-harvesting system for livestock	2.0
		built a water-harvesting system for domestic consumption	1.1

## 4. Discussion

### (a) Farmer vulnerability

Across the regions studied, smallholder farmers live in precarious conditions and are intrinsically vulnerable to any shocks that affect their agricultural systems. As in much of rural Madagascar and other parts of Africa [27–29], the farmers live in rustic houses, lack electricity and running water, own few assets and rely on natural ecosystems for drinking water, firewood, wild foods and materials for household construction. Agriculture is the mainstay of farmer livelihoods, serving both as the primary source of household food and principal means of income generation. Consequently, the fate of these smallholders is closely interwoven with that of farming.

Malagasy farmers are particularly vulnerable to any reductions in crop productivity for a variety of reasons. First, the farmers cultivate very small parcels of land (less than 1 ha), dedicate most of their land to crop production for household consumption and obtain low crop yields, which are insufficient to meet household needs, let alone provide surplus for sale. In focal group discussions, farmers reported obtaining rice yields of only 0.7–0.8 tons ha^–1^, which is even lower than the national (low) average of 2.1 tons ha^–1^ [30]. The low (and declining) yields in our study regions probably reflect the limited use of inputs (fertilizers, pesticides, improved seed varieties), the lack of animal traction, the use of low technology practices, the use of suboptimal land for rice, the prevalence of slash and burn rice production, and land degradation—all of which have been identified as constraints to agricultural productivity elsewhere [27,31].

A majority of households in all three landscapes are chronically food insecure, which makes them extremely vulnerable to any climate or non-climatic shocks that further reduce agricultural production and food availability. Even in normal years, three-quarters of the farming households lack sufficient food to feed their families and spend, on average, 3.8 months without sufficient food. Food pressure is most acute in the months immediately prior to the main rice harvest. This seasonal pattern of food insecurity occurs across the country, with an estimated additional one million Malagasy falling below the poverty threshold during the period of acute food shortage, joining the nine million who are poor year-round [12]. The lack of sufficient food has significant livelihood impacts, including increased rates of malnutrition and child mortality [12].

Another factor that increases farmer vulnerability is the remoteness of farm villages and lack of adequate road infrastructure. Across the three regions, roads are in a poor state and unevenly distributed, with many villages lacking roads that connect them to other villages. Even the main roads are often accessible only during the dry season. The livelihood implications of this isolation are significant, as farmers have difficulties getting their products to markets as well as obtaining agricultural inputs; in addition, farmers generally have to pay higher prices for agricultural inputs in remote areas, reducing their profit margins [31].

A final set of factors that exacerbate farmer vulnerability is that most households lack access to formal safety nets to which they could turn in times of need. Most of the smallholder farmers remain outside a formal credit or banking system, lack capital and are unable to access credit or loans (less than 2% of the farmers surveyed had either a personal savings account or village savings accounts). There are no developed insurance markets and instead farmers rely on informal support systems, borrowing money or food from family or friends. In addition, although there are numerous local NGOs working in the three regions, there is no formal extension service and only 7% of the farmers currently receive any technical support. Farmers are further constrained by having limited access to agrometeorological or market information (only 19% of the households have mobile phones), which could help inform farm management decisions, such as the choice of crops, planting dates and management strategies, and which could serve as early warning systems for floods and cyclones [32].

### (b) Risks and risk coping strategies

In all three regions, smallholder farmers face multiple, recurring and substantial risks to their agricultural production and livelihoods—including risks owing to pest and disease, risks related to weather events and climate change, and those related to market access/price volatility. Farmers routinely face significant pest (particularly mice) and disease outbreaks (particularly rice blast, *Pyricularia oryzae)* and the accompanying crop and income losses, while highly variable, can be substantial (e.g. 15% of farmers reported losing more than half of their crop to pests and diseases).

In addition, farmers are frequently subjected to extreme weather events, which result in crop and livestock losses, as well as damage to agricultural fields, roads and homes. Cyclones are a prominent feature of Madagascar's climate, occurring from November to May, with an average of three to four cyclones per year [9,33]. Cyclones have particularly detrimental impacts on smallholder farmers because the peak cyclone season (January–February) occurs during the ‘lean season’ when farmers are already experiencing food shortages. In addition, cyclones often completely devastate crop yields, leaving farmers without the means to generate income. As in other regions where cyclones are common [34], the recurring nature of cyclones makes it extremely difficult for farmers to move out of poverty, as there is often little time for farmers to rebuild their houses, replant their crops and recover before another cyclone hits.

Farmers are also affected by problems of market access and price volatility. Despite the fact that most farmers in the study regions do not produce enough rice to feed their families, 84% of households sell some of their crop immediately following the harvest to cover the costs of inputs and basic household needs. Later in the year, when their rice reserves run out, these same families typically buy back rice in the market, often at higher prices—a phenomenon that is common across Madagascar [8]. Rice prices are generally the lowest immediately after the harvest, and the highest during the lean season when farmers buy rice back to feed their families [18], thereby reducing the ability of farmers to purchase food. Related problems include difficulties of farmers getting their produce to market, owing to the lack of road infrastructure as well as low demand for some products.

Farmers in all three regions use a variety of coping strategies to deal with impacts on their agricultural production and food security. One of the most common strategies for households is to consume less food or to switch their diet from rice to cassava and other tubers. A subset of farmers also rely heavily on wild foods from communal forests to supplement their diets. Wild yams are particularly valuable for farmers because their harvest season coincides with the period of rice shortage and they can be easily stored for long periods of time once they have been processed [35]. Farmers also find means of generating extra income so that they can purchase food in the market, often selling small livestock (e.g. chickens) or working as agricultural wage labourers on other farms. Last, but not least, farmers turn to relatives or friends for support—to borrow money, obtain help in rebuilding houses or borrowing food. These social relationships are particularly critical given the lack of formal safety nets. Studies of smallholder farmers elsewhere have reported a similar set of coping strategies [4,34,36–38]. However, a few strategies that are common elsewhere—such as receiving food aid, participating in food for work programmes, receiving support from local organizations or migrating to another area—were only rarely reported by farmers in our study regions.

While these coping strategies clearly help to mitigate impacts on farmer livelihoods, the fact that most farmers suffer chronic food insecurity suggests that these coping strategies are insufficient. In addition, there are limits to how much different coping strategies can be successfully used. For example, off-farm employment opportunities are often restricted to the months when fields need to be planted and opportunities may be limited. Harvesting wild yams to supplement food supplies may be unsustainable in the long run, if farmers overharvest them or if the forest ecosystems are degraded. In addition, if all households in a given village are impacted by a cyclone, farmers are unable to turn to neighbours or family members living in the same region to borrow money, as these households will similarly be in need of support. There is therefore an urgent need to provide coping strategies and safety nets, which can better alleviate chronic food insecurity, both in regular years and in times of stress.

### (c) Climate change and adaptation needs

Climate change will likely have significant livelihood impacts on the smallholder farmers in all three regions and further exacerbate food insecurity and poverty. Climate models suggest that Madagascar will experience an increase in mean temperature of 1.1–2.6°C this century, as well as increases in rainfall across the island in summer and increases in rainfall in winter everywhere except the southeast coast [9]. The destructive force of cyclones is also expected to increase [9]. In addition, most climate models show a projected negative impact of climate change on crop productivity in Africa models [39,40]. For example, a recent synthesis of models of the projected impacts of climate change on agriculture indicated that maize and cassava production will be significantly reduced by mid-century (with mean estimates of an aggregate 22% reduction in mean maize yields across sub-Saharan Africa, and an 8% reduction for cassava; [40]). These changes will probably place farmers under additional stress, both owing to direct reductions in agricultural productivity and through impacts on human health, infrastructure and availability of firewood and other ecosystem services on which the poor depend [4,5].

In all three landscapes studied, most farmers reported that they had noticed changes in climatic conditions over the last 10 years, with more than 90% reporting increase in temperature and changes in rainfall patterns. It is not possible for us to determine whether or not these perceived changes are accurate, owing to the lack of long-term climatic data for these landscapes. However, it is clear that farmer's perceptions of climate change, regardless of whether these are correct or not, are already causing some farmers to change their agricultural practices and have important consequences for their livelihoods. Other studies have similarly shown that farmer perceptions of climate change are an important factor driving the adoption of different livelihoods strategies and adaptation measures [29,41,42].

Interestingly, while most Malagasy farmers already perceive the impacts of climate change, as in other parts of Africa [29], only a subset (21%) have changed their farming systems in response to these changes. The limited uptake of adaptation strategies by farmers is probably due to the high levels of household food insecurity, which make it risky for farmers to adopt new strategies that may affect their agricultural production and food availability. In addition, most farmers in our region simply lack the resources needed to implement adaptation measures, as has been found in other regions [29,43]. The fact that the use of adaptation measures was positively correlated with farmer education level, use of diversified agricultural practices, diversified cropping systems and livestock ownership indicate that farmers who are better educated and already have more diversified systems are more likely to be willing to adopt new strategies. Other studies have similarly highlighted the importance of educational level, wealth, access to credit and information, extension services, safety nets, resources and adequate agricultural inputs and technologies in increasing the probability of uptake of adaptation measures by smallholder farmers [29,43,44].

### (d) Policy options for reducing farmer vulnerability in a changing climate

Farmers in our study regions are in a vicious cycle of food insecurity due to low yields, regular shocks that reduce agricultural yields and inadequate coping strategies, and this situation is likely to be further exacerbated by climate change. An inevitable question given the bleak outlook is whether farming is really a viable option for improving farmer livelihoods, or whether policymakers should focus instead on developing alternative employment strategies for these rural populations. In the study areas—and in most rural areas of the country—there are few employment alternatives available to farmers and the poor infrastructure and lack of basic services make it extremely difficult to promote non-farming activities, so farmers will inevitably continue to farm in the absence of other options. In addition, while migration of farmers from rural areas to the urban areas in search of employment does occur, it is unlikely that the cities can successfully absorb the estimated 70% of the population that currently depends on farming for their livelihoods. Efforts to improve the livelihoods of smallholder farmers, therefore, will necessarily need to focus, at least in the near term, on increasing agricultural productivity and making farmer livelihoods less vulnerable to climate change and other risks.

Particular attention must be paid to raising agricultural productivity, as this could make a significant difference in food insecurity and poverty levels, both by increasing the total food availability to households and improving household income generation [2,12]. Agricultural growth has been shown to be 2.2 times as effective at reducing poverty as growth in non-agricultural sectors [45], indicating the critical role that improving agricultural productivity should play in development strategies. Efforts to improve agricultural productivity should target not only rice—the staple of Malagasy diets—but also the production of secondary food crops, such as maize, cassava and other tubers, as these are the foods that the rural poor turn to during the lean season.

There are many potential options that could increase the agricultural production and improve the livelihoods of Malagasy farmers. These range from high-level transformations (such as changes in agricultural policies, economic development policies, poverty reduction strategies, public safety nets, market reforms, institutional arrangements and governance structures; [5,8,12,14,18,27]) to more local-level actions, which aim to directly improve farmer productivity. While systematic and transformational changes in policies and governance are urgently needed to address Madagascar's high poverty and chronic food insecurity, these changes are extremely difficult to achieve and fall beyond the scope of our paper. Our focus here, instead, is on specific technical options, which we believe hold promise as low-cost, feasible and relatively fast opportunities for improving agricultural productivity on farms, which can be pursued even in the context of unfavourable policies and institutional arrangements.

Options that have been shown to be effective in increasing agricultural productivity elsewhere in Africa include facilitating access to improved seed varieties, fertilizers, irrigation and other inputs [31], improving road infrastructure and access to markets [28,38], providing greater technical support and extension services to farmers [29,43], and facilitating access to timely climate information, which could be used to inform the choice of crops, planting dates and management strategies [2,32], among others. Given the high poverty levels in Madagascar and limited public expenditure, some of these options (such as improving the road network in rural areas or increasing the distribution and use of farm inputs), though desirable, are unlikely to be feasible in the short term.

Our research suggests four potential areas for policymakers to pursue that could help to increase agricultural productivity and improve livelihoods in the short term. First, there is an urgent need to improve farmer extension services to provide technical information and training on the best management practices for planting, harvesting and crop storage, to facilitate the adoption of new management practices and to encourage farmer-to-farmer learning. Strengthening extension services has been shown to be particularly effective at convincing farmers to change farming practices in response to climate change [29,43]. Our results show that only 7% of farmers in our study regions currently have access to technical support on agriculture and that the adoption of management practices aimed at reducing vulnerability to climate risks is low, despite the prevalence of these risks. These results indicate that there is significant scope for relatively low-cost farmer extension services to improve the uptake of such practices and provide ongoing technical support. For example, changes in crop planting schedules, management practices and varieties used, as well as the diversification of crops planted, are all low-cost options for reducing agricultural risk, which could be widely promoted through extension services and communication campaigns [46]. Careful screening of these strategies and participatory action-oriented research with farmers will be needed to jointly identify and implement adaptation options that are feasible and effective and to ensure that these strategies do not have any negative or unexpected impacts on farmer livelihoods [46,47].

The second low-cost opportunity for policymakers and donors is to invest in small-scale infrastructure, such as improved irrigation systems or crop storage facilities, which can help farmers to increase production and better protect their harvests. Smallholder farmers in Madagascar are very keen to build local infrastructure but rarely have the necessary capital to finance these activities. There are many examples in Madagascar of grants and even small loans being used to help with such investments that result in important increases in areas under cultivation and agricultural yields [48,49]. Governments and organizations working in remote areas should seek to further promote such small-scale infrastructure through the development of small-scale grants and credit to farmers or local farmer associations.

The third option for improving farmer livelihoods is to increase access to credit and safety nets during lean periods and following catastrophic events, such as extreme weather events or disease and pest outbreaks. In these extreme situations, many farmers currently depend on informal support from families and friends, as formal safety nets are lacking. There is a critical need to establish formal safety nets and also strengthen informal safety networks to ensure that farmers can access support when they need it. In addition, more innovative solutions are needed to facilitate access of farmers to financial services in terms of need. New services, such as mobile telephone payment systems that are beginning to be available even in remote areas, provide an important new, cheap and secure way for family and friends to exchange money even when they are not physically close to each other. Governments should work with the private sector mobile telephone companies to improve mobile coverage and access to such services. Village savings and loans groups in which members pool resources and lend to members in need are also a low-cost solution that could help to reduce the worst impacts of the lean season or extreme weather events, while creating local funds that farmers can tap into for other development activities [50,51].

The final priority for policymakers is to safeguard the natural ecosystems that smallholder farmers use as safety nets. Forests, wetlands, rivers and other natural areas provide critical ecosystem services to Malagasy smallholder farmers, including the provision of firewood and charcoal, water, wild yams and materials for house construction [52,53], among others. These services are important year-round, but particularly following catastrophic events when farmers turn to the forests for food and materials to rebuild their damaged homes. Efforts that conserve, restore or sustainably manage these natural ecosystems are therefore crucial for sustaining farmer livelihoods.

## 5. Conclusion

Our research has highlighted the precarious condition of smallholder farmers in Madagascar, their high exposure to risks and the urgent need to reduce both their current and future vulnerability to these risks. Increasing the productivity and resilience of smallholder farming systems is a huge challenge that will require significant and sustained technical, financial and political support and action at both the national and local levels. However, a handful of low-cost and local approaches—such as revitalizing farmer extension services, implementing small-scale local infrastructure projects with farmers, strengthening informal safety nets and safeguarding natural ecosystems—could go a long way towards beginning to address this critical challenge and improving the livelihoods of smallholder farmers across the country.

## References

[1] Nagayet O 2005 Small farms: current status and key trends. In The future of small farms: proceedings of a research workshop (ed. IFPRI), pp. 355–367. Washington, DC: International Food Policy Research Institute.

[2] Sanchez PA Swaminathan MS 2005 Cutting world hunger in half. Science 307, 357–359. (10.1126/science.1109057) 15661994

[3] O'Brien K 2004 Mapping vulnerability to multiple stressors: climate change and globalization in India. Glob. Environ. Change 14, 303–313. (10.1016/j.gloenvcha.2004.01.001)

[4] Morton JF 2007 The impacts of climate change on smallholder and subsistence agriculture. Proc. Natl Acad. Sci. USA 104, 19 680–19 685. (10.1073/pnas.0701855104) PMC2148357 18077400

[5] Hertel TW Rosch SD 2010 Climate change, agriculture and poverty. Policy Research Working Paper 5468 Washington, DC: World Bank.

[6] McDowell JZ Hess JJ 2012 Accessing adaptation: multiple stressors on livelihoods in the Bolivian highlands under a changing climate. Glob. Environ. Change 22, 342–352. (10.1016/j.gloenvcha.2011.11.002)

[7] Kevan PG 1999 Pollinators as bioindicators of the state of the environment: species, activity and diversity. Agric. Ecosyst. Environ. 71, 325–352.

[8] Minten B Barrett CB 2008 Agricultural technology, productivity and poverty in Madagascar. World Dev. 36, 797–822. (10.1016/j.worlddev.2007.05.004)

[9] Tadross M Randriamarolaza L Rabefitia Z Zheng KY 2008 Climate change in Madagascar; recent past and future. Washington, DC: World Bank.

[10] World Bank. 2012 World Development Indicators. http://data.worldbank.org/data-catalog/world-development-indicators/wdi-2012.

[11] UNDP. 2011 Human development report: sustainability and equity: a better future for all. New York, NY: United Nations Development Program.

[12] Dostie B Haggblade S Randriamamonjy J 2002 Seasonal poverty in Madagascar: magnitude and solutions. Food Policy 27, 493–518. (10.1016/S0306-9192(02)00063-5)

[13] Institut National de la Statistique (INSTAT). 2010 Enquête Démographique et de Santé de Madagascar 2008–2009. Antananarivo, Madagascar: INSTAT.

[14] Zeller M Lapneu C Minten B Ralison E Randrianaivo D Randrianarisoa C 1999 Pathways of rural development in Madagascar: an empirical investigation of the critical triangle between environmental sustainability, economic growth and poverty alleviation. Q. J. Int. Agric. 2, 105–127.

[15] World Bank. 2012 World DATAbank. http://data.worldbank.org/country/madagascar.

[16] Harper GJ Steininger MK Compton JT Juhn D Hawkins F 2007 Fifty years of deforestation and forest fragmentation in Madagascar. Environ. Conserv. 34, 325–333. (10.1017/S0376892907004262)

[17] Styger E Fernandes ECM Rakotondrasamasy HM Rajaobelinirina E 2009 Degrading uplands in the rainforest region of Madagascar: fallow biomass, nutrient stocks, and soil nutrient availability. Agroforestry Syst. 77, 107–122. (10.1007/s10457-009-9225-y)

[18] Barrett CB Dorosh PA 1996 Farmers’ welfare and changing food prices: non parametric evidence from rice in Madagascar. Am. J. Agric. Econ. 78, 656–669. (10.2307/1243283)

[19] Raharinjanahary H Rabeharisoa L Alizany N Ranaivonasy J Rakotondravelo JC Rabarijohn R Ramparany M Tiani AM 2010 Recherche action participative et dynamisme des agriculteurs malgaches face aux changements climatiques: cas de la région d'Analanjirofo (côte est de Madagascar). Madagascar: Projet A.C.C.A http://www.accca.laboradioisotopes.com.

[20] Ministry of Agriculture. 2004 A monography of the Ambatondrazaka region. Ministry of Agriculture of Madagascar, p. 120 http://www.agriculture.gov.mg.

[21] Government of Madagascar, 2013 Arrete no. 9874/2013 modifiant certaines dispositions de l'arrete interministeriel no. 52005/2010 du 20 decembre 2010 modifiant l'arrete interministeriel Mine-Forets no. 16633 du 17 octobre 2008 portant mise en protection temporaire globale des sites vises par l'arrete no. 17914 du 18 octobre 2006 et levant la suspension de l'octroi de permis miniers et forestier pour certains sites. Antananarivo, Madagascar: Government of Madagascar.

[22] Ministry of Agriculture. 2009 Annuaire Agricole 2005–2008. Ministry of Agriculture of Madagascar, p. 112 http://www.agriculture.gov.mg.

[23] Ministry of Agriculture. 2004 A monography of Vatovavy Fito Vinanay, Amoron'ny Mania, Vakinanakaratra, Ambatondrazaka regions. Ministry of Agriculture of Madagascar http://www.agriculture.gov.mg.

[24] Ministry of Agriculture. 2004 A monography of Mahajanga region. Ministry of Agriculture of Madagascar, p. 111 http://www.agriculture.gov.mg.

[25] Di Rienzo JA Casanoves F Balzarini MG Gonzalez L Tablada M Robledo CW 2012 InfoStat version 2012 Grupo InfoStat, FCA. Argentina: Universidad Nacional de Córdoba See http://www.infostat.com.ar.

[26] StataCorp. 2007 Stata statistical software: release 10. College Station, TX: StataCorp LP.

[27] Randrianarisoa JC Minten B 2001 Agricultural production, agricultural land and rural poverty in Madagascar. Cornell Food and Nutrition Program Working Paper 112 New York, NY: Cornell University See 10.2139/ssrn.439101.

[28] Barrett CB Moser CM McHugh OV Barison J 2004 Better technology, better plots or better farmers? Identifying changes in productivity and risk among Malagasy rice farmers. Am. J Agric. Econ. 86, 869–889. (10.1111/j.0002-9092.2004.00640.x)

[29] Bryan E Deressa TT Gbetibouo GA Ringler C 2009 Adaptation to climate change in Ethiopia and South Africa: options and constraints. Environ. Sci. Policy 12, 413–426. (10.1016/j.envsci.2008.11.002)

[30] Roubard F 1997 La question rizicole à Madagascar: les résultats d'une décennie de libéralisation. In Economie de Madagascar (eds Rajobelina P Razafinrakoto M), pp. 37–61. Anatananarivo, Madagascar: Banque Central de Madagascar and Institut National de la Statistique.

[31] Minten B Randrianarisoa JC Barrett CB 2007 Productivity in Malagasy rice systems: wealth-differentiated constraints and priorities. Agric. Econ. 37, 225–237. (10.1111/j.1574-0862.2007.00247.x)

[32] Vogel C O'Brien K 2006 Who can eat information? Examining the effectiveness of seasonal climate forecasts and regional climate-risk management strategies. Clim. Res. 33, 111–222. (10.3354/cr033111)

[33] Government of Madagascar. 2008 Damage, loss and needs assessment for disaster recovery and reconstruction after the 2008 cyclone season in Madagascar. http://www.3adi.org/tl_files/3ADIDocuments/Country%20information/Madagascar/Madagascar_gov_2008_recovery_plan.pdf.

[34] Hahn MB Riderer AM Foster SO 2009 The livelihood vulnerability index: a pragmatic approach to assessing risks from climate variability and change—a case study in Mozambique. Glob. Environ. Change 19, 74–88. (10.1016/j.gloenvcha.2008.11.002)

[35] Ackermann K 2004 Utilization of wild growing yams as supplementary nutrition and its impact on the dry forest ecosystem in north-western Madagascar. Schweiz Z. Forstwes. 155, 80–88. (10.3188/szf.2004.0080)

[36] Jacoby H Skoufias E 1997 Risk, financial markets and human capital in a developing country. Rev. Econ. Stud. 64, 311–335. (10.2307/2971716)

[37] Dercon S Krishnan P 1996 Income portfolios in rural Ethiopia and Tanzania: choices and constraints. J. Dev. Stud. 32, 850–875. (10.1080/00220389608422443)

[38] Dercon S 2002 Income risk, coping strategies and safety nets. The World Bank Research Observer 17, 141–166. (10.1093/wbro/17.2.141)

[39] Challinor A Wheeler T Garforth C Caufurd P Kassam A 2007 Assessing the vulnerability of food crop systems in Africa to climate change. Clim. Change 83, 381–399. (10.1007/s10584-007-9249-0)

[40] Schlenker W Lobell DC 2010 Robust negative impacts of climate change on African agriculture. Environ. Res. Lett. 5, 014010 (10.1088/1748-9326/5/1/014010)

[41] Thomas DSG Tywman C Osbahr H Hewtison O 2007 Adaptation to climate change and variability: farmer responses to intra-seasonal precipitation trends in South Africa. Clim. Change 83, 301–322. (10.1007/s10584-006-9205-4)

[42] Hassan R Nhemachena C 2008 Determinants of African farmer's strategies for adapting to climate change: multinomial choice analysis. Afr. J. Agric. Resour. Econ. 2, 83–104.

[43] Madison D 2007 The perception of and adaptation to climate change in Africa. World Bank Policy Research Working Paper 4308. Washington, DC: The World Bank.

[44] Ziervogel G Bharwani S Downing TE 2006 Adapting to climate variability: pumpkins, people and pumps. Nat. Resour. Forum 30, 294–305. (10.1111/j.1477-8947.2006.00121.x)

[45] Christiansen L Demery L Kühl J 2006 The role of agriculture in poverty reduction: an empirical perspective. World Bank Policy Research Working Paper, vol. 4013, pp. 1–49. Washington, DC: The World Bank.

[46] FAO. 2010 Climate-smart agriculture: policies, practices and financing for food security, adaptation and mitigation. Rome, Italy: Food and Agriculture Organization of the United Nations See http://www.fao.org/docrep/013/i1881e/i1881e00.htm.

[47] Howden SM Soussana JF Tubiello FN Chhetri N Dunlop M Meinke H 2007 Adapting agriculture to climate change. Proc. Natl Acad. Sci. USA 104, 19 691–19 696. (10.1073/pnas.0701890104) PMC2148359 18077402

[48] USAID. 2010 Paradise lost? Lessons from 25 years of USAID Environment Programs in Madagascar. Washington, DC: U.S. Agency for International Development.

[49] The World Bank. 2008 Project Paper on a Proposed Additional Credit in the amount of SDR 18.5 million (US $30 Million Equivalent) to the Republic of Madagascar for the Rural Development Support Project. Washington, DC: The World Bank.

[50] Heltberg R Siegel PB Jorgensen SL 2009 Addressing human vulnerability to climate change: toward a ‘no-regrets’ approach. Glob. Environ. Change 19, 89–99. (10.1016/j.gloenvcha.2008.11.003)

[51] Bhattamishra R Barrett CB 2010 Community-based risk management arrangements: a review. World Dev. 38, 923–932. (10.1016/j.worlddev.2009.12.017)

[52] Thomas DSG Twyman C 2005 Equity and justice in climate change adaptation amongst natural-resource-dependent societies. Glob. Environ. Change 15, 115–124. (10.1016/j.gloenvcha.2004.10.001)

[53] Hannah L 2008 Climate change adaptation for conservation in Madagascar. Biol. Lett. 4, 590–594. (10.1098/rsbl.2008.0270) 18664414 PMC2610084

